# Drug Discovery in Canine Pyometra Disease Identified by Text Mining and Microarray Data Analysis

**DOI:** 10.1155/2023/7839568

**Published:** 2023-04-17

**Authors:** Xin Wang, Guohua Yu

**Affiliations:** ^1^College of Life Science, Longyan University, Longyan, China; ^2^Fujian Provincial Key Laboratory for the Prevention and Control of Animal Infectious Diseases and Biotechnology, Longyan, China; ^3^Key Laboratory of Preventive Veterinary Medicine and Biotechnology (Longyan University), Longyan, China; ^4^Chinese International College, Dhurakij Pundit University, Bangkok, Thailand

## Abstract

Canine pyometra, which is accompanied by bacterial contamination of the dog uterus, is defined as a complex disease associated with the activation of several systems, including the immune system. This study uses text mining and microarray data analysis methods to discover some existing targeted gene drugs and expand potential new drug indications. Text mining (“canine pyometra”) and microarray data analysis (GSE99877) were used to obtain a common set of genes. These genes and protein-protein interaction (PPI) networks were analyzed using Gene Ontology and the Kyoto Encyclopedia of Genes and Genomes. Then, the important genes clustered in the PPI network were selected for gene-drug interaction analysis to provide evidence for potential drug discovery. Through text mining and data analysis, we obtained 17,544 text mining genes (TMGs) and 399 differentially expressed genes (DEGs), respectively. There were 256 repeat genes between TMGs and DEGs, including 70 upregulated genes and 186 downregulated genes. Thirty-seven genes clustered in three significant gene modules. Eight of the 37 genes can target 23 existing drugs. In conclusion, the discovery of 8 immune response-related genes (*BTK*, *CSF2RA*, *CSF2RB*, *ITGAL*, *NCF4*, *PLCG2*, *PTPRC*, and *TOP2A*) targeting 23 existing drugs may expand the drug indications for pyometra-related diseases in dogs.

## 1. Introduction

Pyometra is a commonly occurring uterine disease in female dogs that often leads to loss of breeding potential and can be life-threatening [1]. On average, one in five bitches is diagnosed with the disease before 10 years of age, and the risk of developing pyometra exceeds 50% in certain dog breeds above this age [2]. The pathogenesis of pyometra is not fully understood, but hormonal influence of the uterus in combination with bacterial infection is currently considered to cause the disease [3]. Bacterial infection of the uterus can lead to sepsis and related endotoxemia and organ dysfunctions in severely affected bitches [4]. The most frequent bacterium often isolated from pyometra uteri in female dogs is *Escherichia coli* [5]. Meanwhile, a series of severe subsequent complications of pyometra reported includes sepsis, septic shock, peritonitis, disseminated bacterial infection, and kidney injury [6, 7]. Surgical ovariohysterectomy is the treatment of choice for pyometra and is considered to be safe and effective [8]. To preserve fertility or if surgery or anesthesia is to be avoided, several medical treatment options are available. Drugs available for medical treatment of pyometra include progesterone–receptor antagonists (aglepristone and mifepristone), prostaglandins (dinoprost and cloprostenol), dopamine agonists (cabergoline), or different combinations of these drugs [9–11]. Drug treatment has a great potential developing in restoring clinical and uterine changes.

As a variety of research methods for bioinformatics, text mining and microarray data analysis have been applied to the screening of disease biomarkers, the identification of signaling pathways, and the discovery of new drugs [12–16]. At the same time, researchers have found a series of pathogenesis of canine pyometra by doing experiments [17–19]. Compared with bioinformatics studies in the field of canine oncology, there are fewer studies on canine pyometra disease through text mining and microarray data analysis.

In this study, text mining and microarray data analysis were used to obtain common genes with altered gene expression between dogs with pyometra and normal healthy dogs, and these genes had intergene/intragene correlations. Subsequently, these genes were clustered into proteins and protein interactions (PPI), and important modular genes with more interactions were identified. Finally, drug-gene interactions of modular genes were performed in the Drug-Gene Interaction Database (DGIdb), with a view to finding several potential biomarkers and existing drugs to provide new therapeutic targets for the prevention and treatment of canine pyometra disease.

## 2. Materials and Methods

### 2.1. Text Mining and Microarray Assay Data Analysis

First, text mining was performed using the open-access website pubmed2ensemble (http://pubmed2ensembl.ls.manchester.ac.uk) [20]. The pubmed2ensemble website can retrieve and extract all gene symbols published by PubMed related to the input keyword [21]. The keyword “pyometra in dogs” was entered in pubmed2integrbl, and then, all unreplicated gene sets were extracted, which constituted text mining genes (TMGs).

Second, the microarray assay data GSE99877 was downloaded from the National Center for Biotechnology Information Gene Expression Comprehensive Database (https://www.ncbi.nlm.nih.gov/geo/) and detected using the Affymetrix® Canine Gene 1.1 ST Array. GSE99877 contains the canine uterus with pyometra (*n* = 4) and controls (*n* = 4) [22]. After downloading the GSE99877 expression matrix, convert the probe identification codes (IDs) to gene symbols. For multiple probes corresponding to the same gene, the value with the most significant expression is used as the gene expression value. Non-mRNA probes were discarded. Then, the gene expression values were normalized using the affy package. Linear models for microarray data (limma) is an R package for the analysis of gene expression matrix, specifically the construction of linear models to assess differentially expressed gene expression under designed experimental conditions. Differentially expressed genes (DEGs) were identified between the canine pyometra group and the healthy control group using the limma package constructed by R. Significantly, DEGs with |log2 − fold change (FC)| ≥ 2.0 and adjusted *p* value (adj.*p*.Val) < 0.01 were selected for further analysis. The intersection of TMGs and DEGs was overlapping genes, which are then subjected to further analysis in the next step.

### 2.2. Gene Ontology (GO) and Pathway Enrichment Analyses

Gene Ontology (GO) is based on existing biological knowledge to describe the role of genes and their products in any organism, and it is divided into three separate branches: biological process (BP), cellular component (CC), and molecular function (MF) [23]. Metabolic pathways and gene signaling networks based on existing databases such as KEGG were used to describe pathway enrichment analyses [24]. Use DAVID, a web-accessible program that integrates functional genome annotation and intuitive graphical summaries [25], to view GO and KEGG enrichment of common genes between TMGs and DEGs; *p* values < 0.05 were considered statistically significant.

### 2.3. PPI Network Construction and Module Analysis

The Search Tool for the Retrieval of Interacting Genes (STRING, version 11.5) database was used to retrieve common genes' encoded protein and PPI network information. Using the Search Tool for Retrieval of interaction Genes (STRING, version 11.5) database, the coding protein and PPI network information of common genes were searched. The database contains >67.6 million proteins and 20 billion interactions involving 14,094 organisms [26]. The common genes were uploaded to the STRING database, and an interaction score > 0.4 (medium confidence) was set as the significance threshold. Then, PPI networks were constructed using the Cytoscape software [27].

Molecular complex detection (MCODE) built into the Cytoscape is an automated method for analyzing highly interconnected modules into molecular complexes or clusters [28]. Except for *K*‐core = 7, the relevant parameter standards are all set by default. Functional enrichment analysis was performed on genes shared between TMGs and DEGs, with *p* < 0.05 as a threshold, from which 3 significant gene modules were screened.

### 2.4. Drug-Gene Interaction and Potential Gene Functional Analyses

The Drug-Gene Interaction Database (DGIdb version 4.2.0, http://www.dgidb.org) is an open-source software that supports searching, browsing, and filtering of drug-gene interaction information based on over 30 trusted sources [29]. Modular genes, as potential targets, are pasted into the drug-gene database to search for existing drug compounds. Potential genes matching the drug were obtained, and functional enrichment analysis was performed.

### 2.5. Statistics Analysis

Moderate *t*-test was used to identify common genes between TMGs and DEGs, and Fisher's exact test was used to analyze GO and KEGG enrichment. All statistical analyses were performed using the R version 4.0.2 software.

## 3. Results

### 3.1. Screening for Common Genes

**Figure 1:**
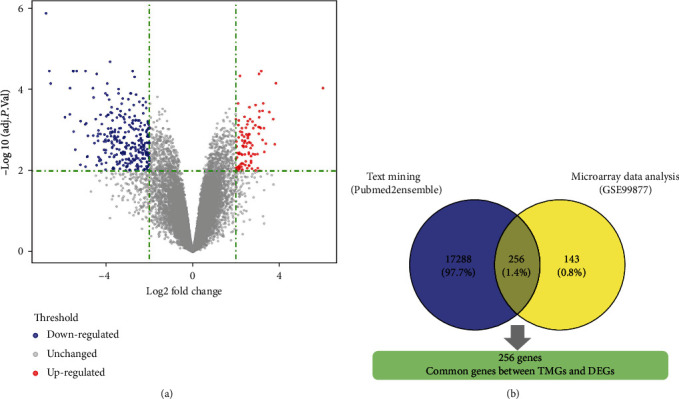
Common genes between TMGs and DEGs. (a) Volcano plot of DEGs. Red: upregulated DEGs; blue: downregulated DEGs. (b) Schematic diagram of screening common genes. Text mining for TMGs and microarray data analysis for DEGs.

Based on the methods of text mining and microarray data analysis, 17,544 TMGs were screened to be associated with canine pyometra. Compared with healthy controls, 399 DEGs were identified in canine pyometra, with 256 genetic overlaps between TMGs and DEGs (Figure 1). Among these overlapping genes were 70 upregulated genes and 186 downregulated genes (Table 1).

### 3.2. GO and Pathway Enrichment Analyses of Common Genes

**Figure 2:**
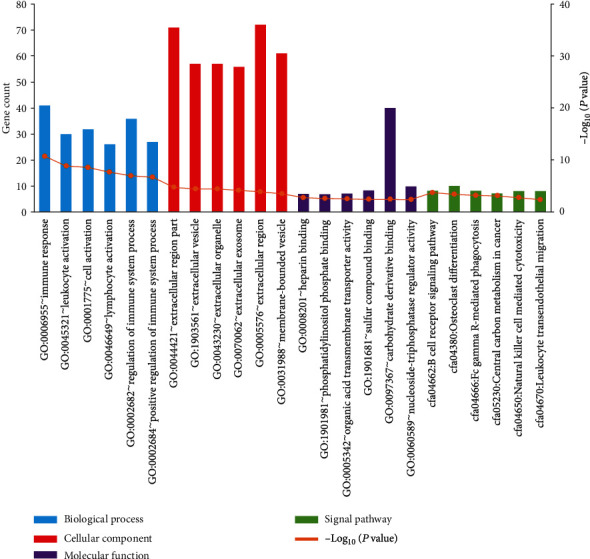
The top six significant GO terms and KEGG pathways of common genes. The bar charts represent the counts of genes classified in the BP, CC, MF, and KEGG, respectively; the yellow line chart represents the significance of enrichment terms. GO: gene ontology; BP: biological process; CC: cellular component; MF: molecular function; KEGG: Kyoto Encyclopedia of Genes and Genomes.

To demonstrate the enrichment of GO and signaling pathways of common genes, functional annotation was performed on the DAVID website. As shown in Figure 2, the top 6 significantly enriched items of the BP, CC, MF, and KEGG signaling pathways of common genes. BP classes were mainly enriched in immune response, leukocyte activation, and cell activation. CC classes were significantly enriched in the extracellular domain fraction, extracellular vesicles, and extracellular organelles. MF classes were mainly enriched in heparin binding, phosphatidylinositol phosphate binding, and organic acid transmembrane transporter activities. In terms of signal pathway enrichment, they are mainly involved in the B cell receptor signaling pathway, osteoclast differentiation, and Fc gamma R-mediated phagocytosis (Figure 2, Table 2).

### 3.3. PPI Network and Module Analysis of Common Genes

**Figure 3:**
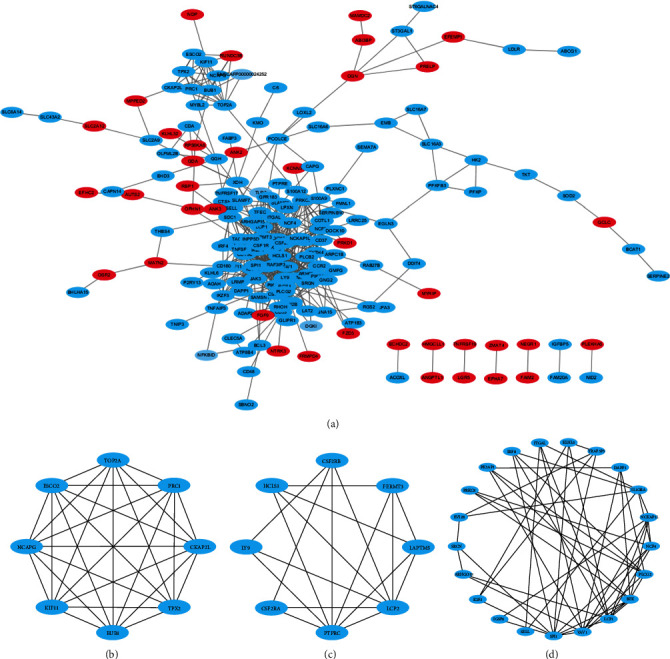
The protein-protein interaction (PPI) network construction and significant gene module analysis; red indicates upregulated genes and light blue indicates downregulated genes. Analysis was performed with MCODE. (a) The entire PPI networks of common genes; (b) module 1 consists of 8 nodes/genes; (c) module 2 consists of 8 nodes/genes; (d) module 3 consists of 21 nodes/genes.

All common genes were pasted on the STRING website and analyzed with the Cytoscape software. A total of 181 genes/nodes, 566 edges participated in the construction of the PPI network, and 75 genes did not appear in the PPI network (Figure 3(a)). The three most significant gene modules were selected using the MCODE application. Module 1 consists of 8 genes/nodes with a total of 28 edges/interactions (Figure 3(b)); module 2 consists of 8 genes/nodes with a total of 18 edges/interactions (Figure 3(c)); module 3 consists of 21 genes/node composition, with a total of 51 edges/interactions, and all showed downregulation (Figure 3(d)).

### 3.4. Drug-Gene Interaction and Functional Enrichment Analysis of Candidate Gene

**Figure 4:**
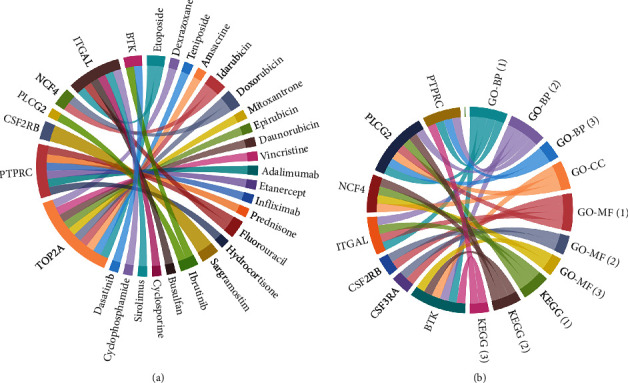
The drugs targeted to genes and their functional enrichment analysis. (a) Chord plot for the connection between 23 drugs and 8 genes; (b) chord plot for functional enrichments of 8 genes. GO: gene ontology; BP: biological process; CC: cellular component; MF: molecular function; KEGG: Kyoto Encyclopedia of Genes and Genomes. GO-BP (1) indicates immune response, GO-BP (2) indicates the cell surface receptor signaling pathway, GO-BP (3) indicates the T cell receptor signaling pathway, GO term-CC indicates cytosol, GO-MF (1) indicates signal transducer activity, GO-MF (2) indicates cytokine receptor activity, GO-MF (3) indicates phosphatidylinositol phosphate binding, KEGG (1) indicates leukocyte transendothelial migration, KEGG (2) indicates osteoclast differentiation, and KEGG (3) indicates primary immunodeficiency.

Thirty-seven cluster genes from significant gene modules were selected for drug-gene interaction analysis. A total of 8 candidate genes were identified targeting 23 potential existing drugs, mainly divided into two drug-gene interaction types (inhibitors and agonists), both of which had initial drug indications (Figure 4(a), Table 3). In addition, the functional enrichment items of these 8 candidate genes mainly involved 7 GO items and 3 KEGG pathways (Figure 4(b), Table 4). The most significant GO items were immune response (BP, *p* value = 9.32*E* − 04), cytoplasm (CC, *p* value = 5.66*E* − 02), and signal transduction activity (MF, *p* value = 4.54*E* − 02). The most significant KEGG pathway was the leukocyte transendothelial migration (*p* value = 4.19*E* − 03).

## 4. Discussion

Cystic endometrial hyperplasia (CEH), mucometra, and pyometra are common uterine diseases in intact bitches, with pyometra being a life-threatening disease usually caused by the hormonal influence of the uterus in combination with bacterial infection [1, 12, 15, 17]. Pyometra, which is accompanied by bacterial contamination of the uterus, is defined as a complex disease associated with the activation of several organ defense responses, including the immune response [4, 5]. It is generally treated with ovariohysterectomy, but several conservative medical options are available [8]. Common drug treatment strategies in combination with antimicrobials are progesterone receptor blockers, which control hormone levels [9–11]. After using a microarray assay, Bukowska et al.'s team identified 17,138 differentially expressed transcripts in the mongrel bitch uterus with pyometra [22]. A total of 264 genes were related to the inflammatory response, 98 of which increased in the expression, 10 decreased, and the remaining were unchanged. It is important to be aware that some complications and organ dysfunctions in pyometra are not associated with systemic inflammation, and its specific molecular biological mechanism is still unclear. Therefore, an in-depth study of the key regulatory genes and drug discovery of signaling pathways is of great significance for the diagnosis and treatment of canine pyometra. Meanwhile, bioinformatics, as an emerging discipline, is used to deal with medical data to identify novel diagnosis markers and discover new drugs [12–14]. However, to date, no researchers have adopted this method to study the drug discovery of canine pyometra.

In this study, we expected to find out the key candidate genes and signal pathway in canine pyometra. Firstly, we applied text mining to identify 17,544 TMGs that were related to canine pyometra. At the same time, we use microarray data analysis to identify 399 DEGs in the dog uterus with the pyometra group compared with the control dog uterus group. Although using the same data package (GSE99877) for DEG analysis, we reduced the *p* value from 0.05 to 0.01 under the consistent screening conditions compared with Bukowska et al.'s team study [22]. We chose the 256 overlapping genes between TMGs and DEGs including 70 upregulated genes and 186 downregulated genes. Then, we performed GO and KEGG annotation analyses for common genes. Subsequently, a DEG PPI network was constructed, 181 nodes/genes were identified with 566 edges, and the three most significant modules were chosen by using the MCODE application from the PPIs. Finally, we have found eight out of 37 significant module genes that target 23 existing potential drugs which might be used for the treatment of canine pyometra disease.

In our study, we have identified 23 drugs, which have a target to eight downregulated genes (*BTK*, *CSF2RA*, *CSF2RB*, *ITGAL*, *NCF4*, *PLCG2*, *PTPRC*, and *TOP2A*). Pyometra is a uterine disease, which may be associated with an increased immune response against bacterial infections of the uterine fluid as well as a proinflammatory response. Doing bacteriological culture, Bukowska et al.'s team study reported bacterial species including 78% *Escherichia coli*, 13% *Staphylococcus* spp., 4.5% *Streptococcus* spp., and 4.5% *Pseudomonas aeruginosa* in endometrium in dogs severely affected by pyometra [22]. Peter et al. showed that the cow endometrial mRNA expression of protein tyrosine phosphatase receptor type C (*PTPRC*) was lower in the subclinical endometritis *L. buchneri* group compared with the placebo group [30]. Yang et al. reported a cocktail of minichromosome maintenance protein 2 (*MCM2*) and topoisomerase (DNA) II alpha (*TOP2A*), *p16INK4a*, and *Ki-67* as biomarkers for the improved diagnosis of cervical intraepithelial lesion [31]. These significantly downregulated genes have been reported in human beings and animal uterine diseases, which are involved in immune and proinflammatory responses. Therefore, these genes have the potential to be new biomarkers for pyometra in dogs.

In the KEGG pathway group, the downregulated genes were most significantly enriched for the B cell receptor signaling pathway. Bruton's tyrosine kinase (*BTK*) is an important signal molecule of the B cell receptor pathway, expressed in various developmental stages of B cells, participating in regulatory B cell proliferation, differentiation, and apoptosis and playing an important role in the survival and proliferation of malignant B cells [32]. It is the focus of the research on B cell tumors and B cell immune diseases. Launched in 2013, ibrutinib is the world's first commercially available BTK inhibitor and has been approved by FDA for 6 indications: chronic lymphocytic leukemia, small lymphocytic lymphoma, mantle cell lymphoma, Waldenstrom's macroglobulinemia, graft-versus-host disease, and marginal zone lymphoma [32–34]. Ko et al. reported primary lymphoma involving the uterine horn in a 9-year-old intact female Lhasa Apso dog, which was diagnosed as having extranodal marginal zone B cell lymphoma (*MZBCL*) [35]. Therefore, ibrutinib as a BTK inhibitor may be used as a prophylactic or therapeutic agent for canine pyometra caused by B cell lymphoma.

Neutrophils, which are the most abundant leukocytes, migrate from the bloodstream into sites of inflammation in different tissues [36]. These blood cells are the first line of innate immune defense against invading bacteria [37]. After firmly adhering to the endothelium, neutrophils cross this cell barrier and reach the tissue at the site of infection. There, neutrophils by means of phagocytosis, degranulation, and releasing their DNA in response to infectious agents form neutrophil extracellular traps (*NETs*) to destroy pathogens [17, 36]. Conversely, several proinflammatory cytokines, including granulocyte macrophage-colony-stimulating factor (*GM-CSF*), prolong neutrophil survival [38]. Colony-stimulating factor 2 receptor alpha (*CSF2RA*) protein encoded by this gene is the alpha subunit of the heterodimeric receptor for colony-stimulating factor 2, a cytokine which controls the production, differentiation, and function of granulocytes and macrophages [39]. Colony-stimulating factor 2 receptor beta (*CSF2RB*) protein encoded by this gene is the common beta chain of the high-affinity receptor for *IL-3*, *IL-5*, and *CSF* [40]. As a granulocyte-macrophage colony-stimulating factor receptor agonist, sargramostim may be stimulating upregulated expression of *CSF2RA* and *CSF2RB* and activating mature granulocytes and mononuclear macrophages to improve anti-infection and immune function in canine pyometra.

The downregulated genes *ITGAL*, *NCF4*, and *PLCG2* were all significantly enriched for leukocyte transendothelial migration in the KEGG pathway analysis. Tometten et al. provided that stress-triggered abortion is mediated by adhesion molecules, i.e., intercellular adhesion molecule 1 (*ICAM1*) and leukocyte function-associated molecule 1 (*ICAM1*), now being referred to as integrin alpha L (*ITGAL*), which facilitate the recruitment of inflammatory cells to the fetomaternal interface [41]. As immunosuppressive agents, cyclosporine and sirolimus are expected as *ITGAL* gene targets to suppress inflammation caused by pyometra.

Neutrophil cytosolic factor 4 (*NCF4*) protein encoded by this gene is a cytosolic regulatory component of the superoxide-producing phagocyte NADPH oxidase, a multicomponent enzyme system important for host defense [42]. Severe congenital neutropenia syndrome 4, also known as glucose-6-phosphatase-*β* deficiency, is characterized not only by neutropenia but also by impaired neutrophil energy homeostasis and functionality [43]. Jun et al. have demonstrated that the expression of NADPH oxidase subunits and membrane translocation of p47(*phox*) is downregulated, and NCF4 (-/-) macrophages exhibit repressed trafficking in vivo both during an inflammatory response and in pregnancy [44]. 1-phosphatidylinositol 4,5-bisphosphate phosphodiesterase *γ*-2 (*PLCG2*) is an important regulator of embryonic cerebral cortices and osteoclast development by mediating integrin receptor signaling [45]. With interactive network analysis of the placental cotyledon during late pregnancy, Yan et al. reported that upregulated protein *PLCG2* was related to placenta formation, blood flow regulation, and embryonic development [46]. As antineoplastic agents, idarubicin, doxorubicin, and ibrutinib regulated the expression of *NCF4* and *PLCG2* in several recent studies [47–49]. The high incidence of pyometra in old dogs may be related to metabolic disorder and the abnormal expression of protooncogenes. In particular, tumor diseases seriously affect the immune response of elderly pets [2]. Thus, antineoplastic drugs may become preventive or adjuvant therapeutic drugs of pyometra.

In this study, the interaction between the drugs and genes we discovered was mainly divided into two types, namely, agonist and inhibitor. These drugs are mostly classified into anti-inflammatory, antineoplastic, immunosuppressive, and immunomodulating agents. Although these existing drugs provide a new perspective for us to study canine pyometra disease, further clinical trials need to be performed for confirmation of its new function and indications.

## 5. Conclusions

Based on text mining (keywords: canine pyometra) and microarray data analysis (dataset: GSE99877), we found 23 FDA-approved existing drugs targeting 8 genes involved in immune responses. These genes may be used in canine pyometra, as well as its initial drug indications.

## Figures and Tables

**Table 1:** The 256 differentially expressed genes were identified between TMGs and DEGs (GSE99877), including 70 upregulated genes and 186 downregulated genes in dog uterus with pyometra samples compared to control dog uterus samples. (The differentially expressed genes were ranked from the smallest to the largest of adjusted *p* value).

DEGs	Genes
Upregulated	*DGKI*, *SPTLC3*, *FREM2*, *CNTLN*, *DACH1*, *MPPED2*, *SNCAIP*, *FAIM2*, *ZMAT4*, *TNFRSF19*, *PLEKHA5*, *TMOD2*, *PAQR5*, *FGF9*, *MATN2*, *HECTD2*, *TMEM55A*, *ABI3BP*, *OSR2*, *RYR3*, *STOX1*, *FAM149A*, *PRELP*, *PRKD1*, *EFEMP1*, *NFIX*, *ANK2*, *FZD3*, *SESTD1*, *ABCA9*, *OGN*, *RPS6KA6*, *RWDD3*, *SPAG16*, *BCL9*, *DDIT4L*, *OPHN1*, *NTRK3*, *LRCH2*, *NDP*, *FRMPD4*, *MYRIP*, *EFHC2*, *PDE11A*, *PTCH1*, *RUNDC3B*, *NAALADL2*, *PTGIS*, *CACHD1*, *NEGR1*, *RBP1*, *HMGCLL1*, *GABRA3*, *KLHL32*, *RASSF6*, *LGR5*, *ECHDC2*, *GDA*, *ZNF521*, *RCAN2*, *GCLC*, *MAMDC2*, *SLC2A12*, *EPHA7*, *ASPA*, *ANGPTL1*, *AUTS2*, *KCNN3*, *ATRNL1*, and *ANK3*
Downregulated	*NCKAP1L*, *SLC46A3*, *LRMP*, *GGH*, *KLHL6*, *BCAT1*, *FAR2*, *TPX2*, *SLC26A4*, *RGS2*, *OLFML2B*, *RND1*, *KIF11*, *SLC39A14*, *MYBL2*, *LOXL2*, *STK17A*, *SLAMF7*, *LCP1*, *ESCO2*, *CD48*, *PIK3AP1*, *TRAF3IP3*, *ARHGDIB*, *IKZF1*, *PIK3CG*, *DKK2*, *FAM20A*, *ST3GAL1*, *SLC16A6*, *TNIP3*, *RGS1*, *NCF2*, *CECR1*, *AOAH*, *GNG2*, *DAPP1*, *EDEM1*, *LPXN*, *PTPRE*, *IRF4*, *CLEC5A*, *EDIL3*, *SEMA7A*, *BTG2*, *PLAC8*, *GIMAP6*, *NID2*, *ABCG1*, *PDK1*, *ADAM28*, *BHLHA15*, *SPI1*, *BPI*, *SDC1*, *TFEC*, *LCP2*, *LAT2*, *SELL*, *DDX43*, *RAB27B*, *PRKCB*, *INPP5D*, *FFAR2*, *FCGBP*, *CCR2*, *PLCG2*, *CHI3L1*, *TFPI2*, *EVI2B*, *CDH3*, *ATP8B4*, *GMFG*, *IKZF3*, *SLCO2B1*, *SAMSN1*, *SLC16A3*, *CD37*, *P2RY13*, *ARHGAP15*, *INHBB*, *TKT*, *RHOH*, *KMO*, *CSF2RA*, *TNFSF13B*, *MALL*, *CASC5*, *RNASE6*, *SLC1A5*, *TMEM150B*, *ATP13A4*, *TYROBP*, *IRF8*, *ASF1B*, *C6*, *CYTH4*, *CAPN14*, *COTL1*, *SBNO2*, *CD3D*, *ARPC1B*, *CKAP2L*, *PTPRC*, *HCLS1*, *LY9*, *STEAP4*, *PFKP*, *CD180*, *SLAMF6*, *CD79B*, *SLC43A2*, *PFKFB3*, *IGSF6*, *BUB1*, *LAPTM5*, *GNA15*, *FAM78A*, *THBS4*, *AIF1*, *EHD3*, *SERPINB10*, *SLC2A9*, *ATP1B3*, *DOCK2*, *SMPDL3A*, *NDUFA4L2*, *CPM*, *CTSH*, *TNFAIP3*, *TNFRSF17*, *LRRC25*, *TOP2A*, *CPA3*, *XDH*, *SRGN*, *IGFBP5*, *DDIT4*, *CD53*, *ACOXL*, *CDA*, *JAK3*, *S100A12*, *S100A9*, *CSF2RB*, *SLC6A14*, *DHRS9*, *TBX20*, *SERPINE2*, *SPN*, *CAPG*, *PRC1*, *SOD2*, *ST6GALNAC4*, *SLC16A7*, *NCAPG*, *NCF4*, *TMEM45B*, *BTK*, *IL10RA*, *LOC608320*, *ADAP2*, *CSF1R*, *LDLR*, *HK2*, *FABP3*, *ITGAL*, *SLC1A4*, *PCOLCE*, *FAM46C*, *FYB*, *GLIPR1*, *VAV1*, *EMB*, *POU2AF1*, *BCL3*, *TNFSF11*, *FERMT3*, *GPR183*, *FMNL1*, *TAGAP*, *TLR8*, *PLCB2*, *DOCK10*, *PLXNC1*, and *EGLN3*

**Table 2:** The top three gene ontology and pathway enrichment terms of upregulated and downregulated genes, respectively.

Terms	Category	Description	Count	*p* value
Upregulated genes				
GO:0072661	BP	Protein targeting to plasma membrane	3	0.002285
GO:0043583	BP	Ear development	5	0.005787
GO:0007423	BP	Sensory organ development	7	0.007154
GO:0031012	CC	Extracellular matrix	7	0.001358
GO:0045202	CC	Synapse	7	0.003773
GO:0043197	CC	Dendritic spine	3	0.007865
GO:0008201	MF	Heparin binding	4	0.003801
GO:0005539	MF	Glycosaminoglycan binding	4	0.009789
GO:0030507	MF	Spectrin binding	2	0.010964
cfa05205	KEGG	Proteoglycans in cancer	4	0.037887
Downregulated genes				
GO:0006955	BP	Immune response	41	4.12E-16
GO:0045321	BP	Leukocyte activation	30	5.74E-13
GO:0001775	BP	Cell activation	32	7.49E-13
GO:0044421	CC	Extracellular region part	58	5.52E-06
GO:1903561	CC	Extracellular vesicle	47	1.26E-05
GO:0043230	CC	Extracellular organelle	47	1.28E-05
GO:0005342	MF	Organic acid transmembrane transporter activity	7	6.97E-04
GO:0008047	MF	Enzyme activator activity	11	0.002017
GO:0046943	MF	Carboxylic acid transmembrane transporter activity	6	0.003604
cfa04662	KEGG	B cell receptor signaling pathway	8	3.67E-05
cfa04380	KEGG	Osteoclast differentiation	10	5.64E-05
cfa04666	KEGG	Fc gamma R-mediated phagocytosis	8	1.43E-04

Note: GO: gene ontology; BP: biological process; CC: cellular components; MF: molecular function; KEGG: Kyoto Encyclopedia of Genes and Genomes.

**Table 3:** The specified information of drugs and their target genes.

Number	Gene	Drug	Interaction	Drug class^∗^
1	BTK	Ibrutinib	Inhibitor	Antineoplastic agents
2	BTK	Dasatinib	Other	Antineoplastic agents
3	CSF2RA	Sargramostim	Agonist	Immunomodulatory agents
4	CSF2RB	Sargramostim	Agonist	Immunomodulatory agents
5	ITGAL	Busulfan	Other	Not available
6	ITGAL	Etoposide	Other	Not available
7	ITGAL	Fluorouracil	Other	Not available
8	ITGAL	Cyclosporine	Other	Immunosuppressive agents
9	ITGAL	Sirolimus	Other	Immunosuppressive agents
10	ITGAL	Cyclophosphamide	Other	Not available
11	NCF4	Idarubicin	Other	Antineoplastic agents
12	NCF4	Doxorubicin	Other	Antineoplastic agents
13	PLCG2	Ibrutinib	Other	Antineoplastic agent
14	PTPRC	Adalimumab	Other	Anti-inflammatory agent
15	PTPRC	Etanercept	Other	Immunomodulatory agents, anti-inflammatory agent
16	PTPRC	Infliximab	Other	Immunosuppressive agents, anti-inflammatory agent
17	PTPRC	Prednisone	Other	Anti-inflammatory agent
18	PTPRC	Fluorouracil	Other	Not available
19	PTPRC	Hydrocortisone	Other	Not available
20	TOP2A	Etoposide	Inhibitor	Antineoplastic agents
21	TOP2A	Dexrazoxane	Inhibitor	Not available
22	TOP2A	Teniposide	Inhibitor	Antineoplastic agents
23	TOP2A	Amsacrine	Inhibitor	Not available
24	TOP2A	Idarubicin	Inhibitor	Antineoplastic agents
25	TOP2A	Doxorubicin	Inhibitor	Antineoplastic agents
26	TOP2A	Mitoxantrone	Inhibitor	Antineoplastic agents
27	TOP2A	Epirubicin	Inhibitor	Antineoplastic agents
28	TOP2A	Daunorubicin	Inhibitor	Antineoplastic agents
29	TOP2A	Vincristine	Other	Antineoplastic agents

Note: ^∗^the drug indications have been approved by FDA. BTK: Bruton's tyrosine kinase; CSF2RA: colony-stimulating factor 2 receptor alpha; CSF2RB: colony-stimulating factor 2 receptor beta; ITGAL: integrin subunit alpha L; NCF4: neutrophil cytosolic factor 4; PLCG2: phospholipase C-gamma 2; PTPRC: protein phosphatase; TOP2A: topoisomerase II alpha.

**Table 4:** The functional enrichments of the final 8 genes.

Category	ID	Term	Genes	*p*-value
GO-BP	GO:0006955	Immune response	*PTPRC*, *BTK*, *PLCG2*, and *ITGAL*	0.000932
GO-BP	GO:0007166	Cell surface receptor signaling pathway	*PTPRC*, *BTK*, *PLCG2*, and *ITGAL*	0.007812
GO-BP	GO:0050852	T cell receptor signaling pathway	*PTPRC* and *PLCG2*	0.017121
GO-CC	GO:0005829	Cytosol	*NCF4*, *BTK*, and *PLCG2*	0.056613
GO-MF	GO:0004871	Signal transducer activity	*PLCG2*, *CSF2RB*, *ITGAL*, and *CSF2RA*	0.045474
GO-MF	GO:0004896	Cytokine receptor activity	*CSF2RB* and *CSF2RA*	0.04673
GO-MF	GO:1901981	Phosphatidylinositol phosphate binding	*NCF4* and *BTK*	0.054916
KEGG pathway	cfa04670	Leukocyte transendothelial migration	*NCF4*, *PLCG2*, and *ITGAL*	0.004198
KEGG pathway	cfa04380	Osteoclast differentiation	*NCF4*, *BTK*, and *PLCG2*	0.005013
KEGG pathway	cfa05340	Primary immunodeficiency	*PTPRC* and *BTK*	0.030721

Note: GO: gene ontology; BP: biological process; CC: cellular components; MF: molecular function; KEGG: Kyoto Encyclopedia of Genes and Genomes.

## Data Availability

The microarray assay data GSE99877 supporting this article is from previously reported studies and datasets, which have been cited. The data used to support the findings of this study are available from the corresponding author upon request.
